# Supplementary figures and images for: Bactericidal Permeability-Increasing Protein (BPI) Inhibits Mycobacterium tuberculosis Growth
Source: Biomolecules. 2024 Apr 13;14(4):475. doi: 10.3390/biom14040475 (PMC11048543; doi:10.3390/biom14040475)

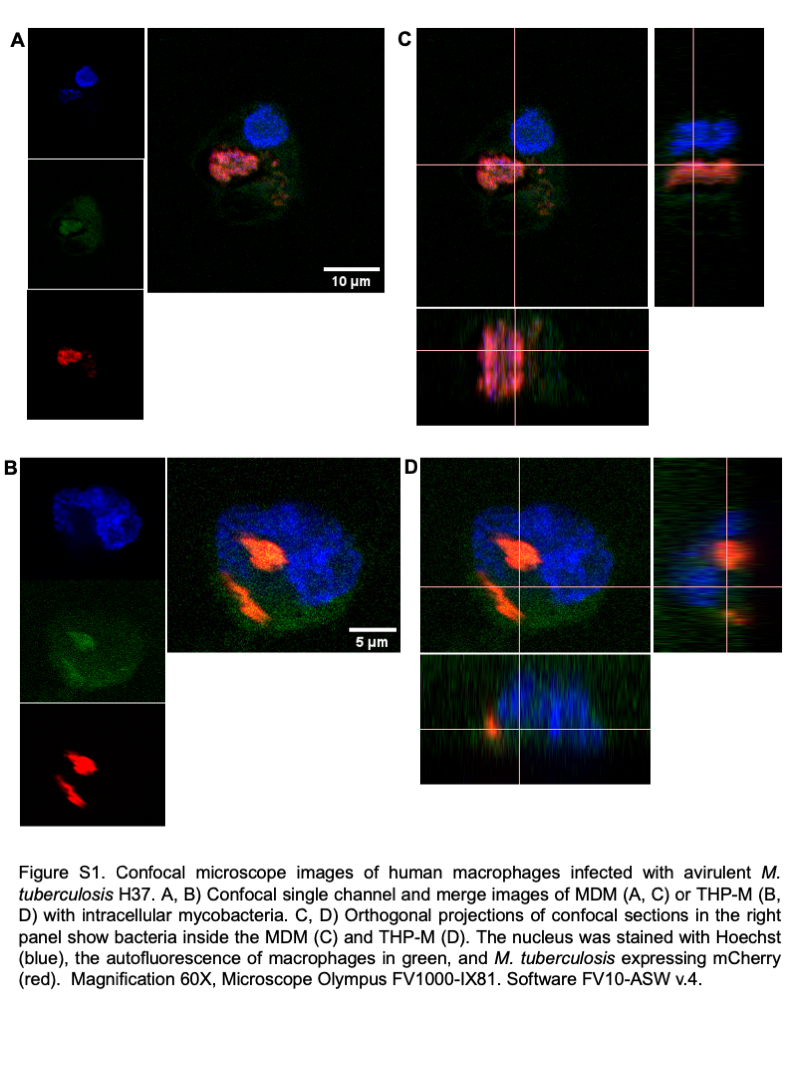

Supplement: Supplementary file 1 [file biomolecules-14-00475-s001.zip › Figure S1.tiff]

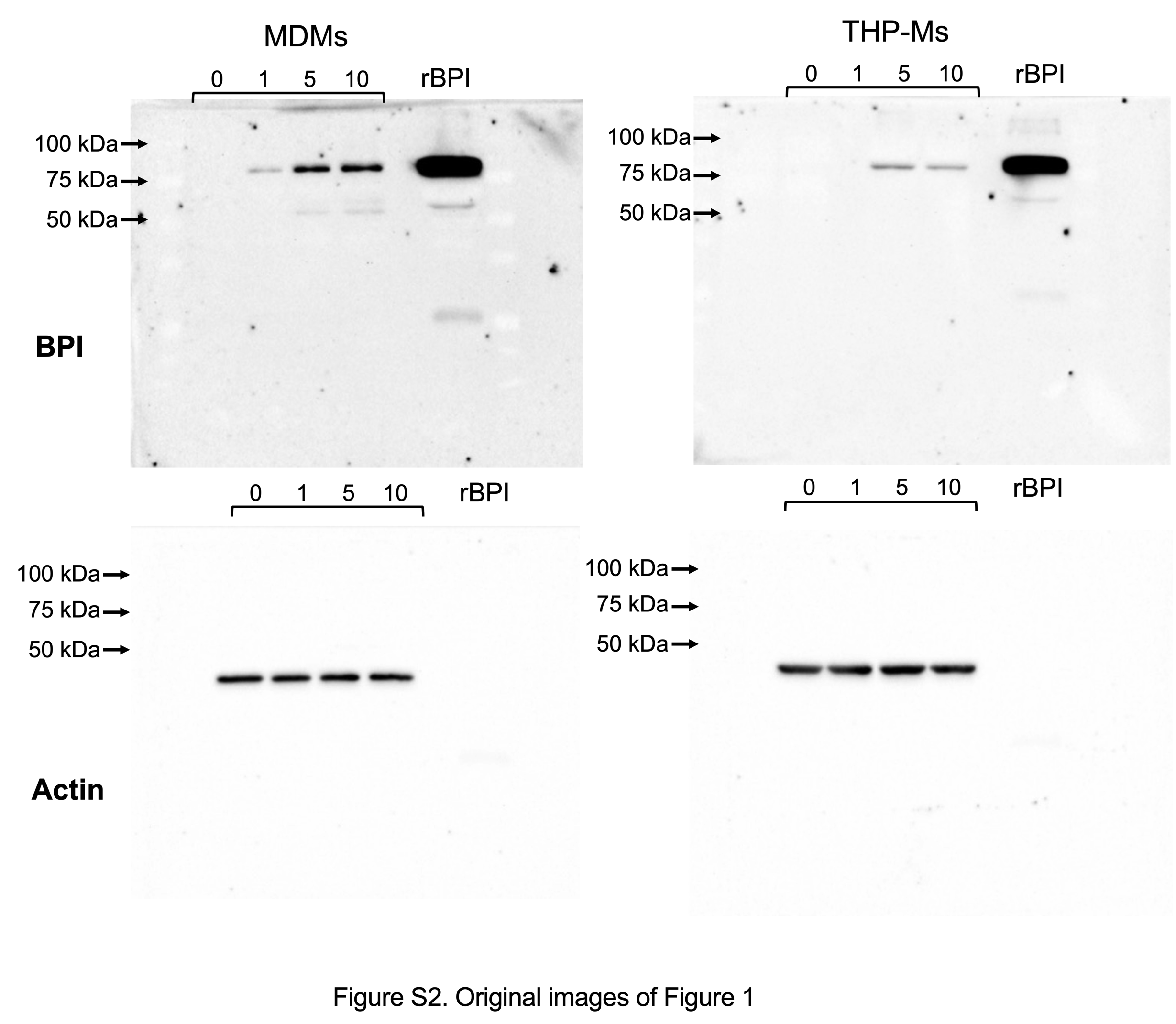

Supplement: Supplementary file 1 [file biomolecules-14-00475-s001.zip › Figure S2.tiff]
